# Author Correction: A morphological analysis of activity-dependent myelination and myelin injury in transitional oligodendrocytes

**DOI:** 10.1038/s41598-021-96317-4

**Published:** 2021-08-18

**Authors:** Eszter Toth, Sayed Muhammed Rassul, Martin Berry, Daniel Fulton

**Affiliations:** 1 Neuroscience and Ophthalmology Research Group, Institute of Inflammation and Ageing, College of Medical and Dental Sciences, University of Birmingham, Edgbaston, Birmingham, B15 2TT UK; 2 School of Psychology, College of Life and Environmental Sciences, University of Birmingham, Birmingham, UK; 3 Physical Sciences of Imaging in the Biomedical Sciences Training Programme, University of Birmingham, Birmingham, UK

Correction to: *Scientific Reports* 10.1038/s41598-021-88887-0, published online 05 May 2021

The original version of this Article contained errors in Figure 5, where data and statistical results shown in panels C and D were based on a previous analysis which did not reflect the aggregation of image data points to individual brain slices.

As a result, the legend of Figure 5

“Sustained inhibition of neuronal activity reduces myelination of cerebellar white matter. (**A**,**B**) Maximum projection images from representative control (**A**) and TTX (**B**) treated cerebellar slices showing immunofluorescent signals for anti-NF200 (**Ai**,**Bi**) anti-MBP (**Aii**,**Bii**), and co-localisations used to quantify myelination (**Aiii**,**Biii**). (**C**) Quantification of anti-NF200 signals. Mean NF200 pixel fraction is reduced by TTX (Control 20.4 ± 0.5, TTX 17.7 ± 0.5). (**D**) Average MBP/NF200 ratios normalized against the NF200 signal are significantly reduced by TTX (Control 0.4 ± 0.02, TTX 0.29 ± 0.02). Scale bars in (**A**) and (**B**) 20 µm. * and *** Significance *P* < 0.05 and *P* < 0.001, respectively. Data expressed as means ± SEM.”

now reads:

“Sustained inhibition of neuronal activity reduces myelination of cerebellar white matter. (**A**,**B**) Maximum projection images from representative control (**A**) and TTX (**B**) treated cerebellar slices showing immunofluorescent signals for anti-NF200 (**Ai**, **Bi**) anti-MBP (**Aii**, **Bii**), and co-localisations used to quantify myelination (**Aiii**, **Biiii**). (**C**) Quantification of anti-NF200 signals. Mean NF200 pixel fraction is reduced by TTX (Control 20.4 ± 0.7, TTX 17.9 ± 0.7). (**D**) Average MBP/NF200 ratios normalized against the NF200 signal are significantly reduced by TTX (Control 0.4 ± 0.02, TTX 0.29 ± 0.02). Scale bars in (**A)** and (**B)** 20 µm. * and ** Significance *P* < 0.05 and *P* < 0.01, respectively. Data expressed as means ± SEM.”

In addition, the part label B has been corrected to ‘Bi’.

The original Figure 5 and its accompanying legend appear below.

**Figure 5:**
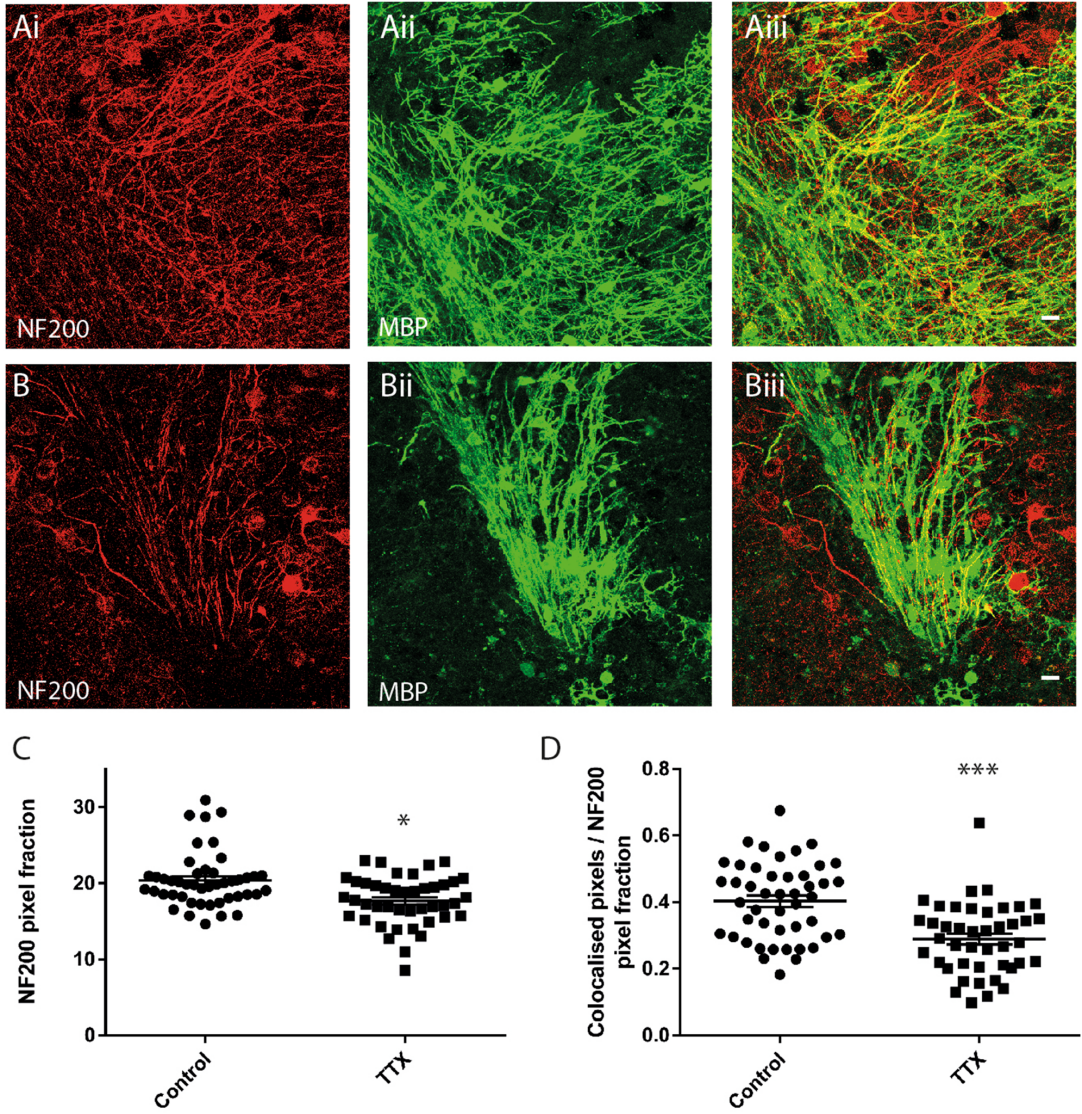
Sustained inhibition of neuronal activity reduces myelination of cerebellar white matter. (**A**,**B**) Maximum projection images from representative control (**A**) and TTX (**B**) treated cerebellar slices showing immunofluorescent signals for anti-NF200 (**Ai**,**Bi**) anti-MBP (**Aii**,**Bii**), and co-localisations used to quantify myelination (**Aiii**,**Biii**). (**C**) Quantification of anti-NF200 signals. Mean NF200 pixel fraction is reduced by TTX (Control 20.4 ± 0.5, TTX 17.7 ± 0.5. (**D**) Average MBP/NF200 ratios normalized against the NF200 signal are significantly reduced by TTX (Control 0.4 ± 0.02, TTX 0.29 ± 0.02). Scale bars in (**A**) and (**B**) 20 µm. * and *** Significance *P* < 0.05 and *P* < 0.001, respectively. Data expressed as means ± SEM.

The original Article has been corrected.

